# Drug Delivery Systems for Hedgehog Inhibitors in the Treatment of SHH-Medulloblastoma

**DOI:** 10.3389/fchem.2021.688108

**Published:** 2021-06-07

**Authors:** Miriam Caimano, Ludovica Lospinoso Severini, Elena Loricchio, Paola Infante, Lucia Di Marcotullio

**Affiliations:** ^1^ Department of Molecular Medicine, University La Sapienza, Rome, Italy; ^2^ Center for Life Nano Science@Sapienza, Istituto Italiano di Tecnologia, Rome, Italy; ^3^ Laboratory Affiliated to Istituto Pasteur Italia-Fondazione Cenci Bolognetti, Rome, Italy

**Keywords:** medulloblastoma, hedgehog signaling, SMO antagonists, GLI inhibitors, drug delivery, nanoparticles, blood–brain barrier

## Abstract

Medulloblastoma (MB) is a highly aggressive pediatric tumor of the cerebellum. Hyperactivation of the Hedgehog (HH) pathway is observed in about 30% of all MB diagnoses, thereby bringing out its pharmacological blockade as a promising therapeutic strategy for the clinical management of this malignancy. Two main classes of HH inhibitors have been developed: upstream antagonists of Smoothened (SMO) receptor and downstream inhibitors of GLI transcription factors. Unfortunately, the poor pharmacological properties of many of these molecules have limited their investigation in clinical trials for MB. In this minireview, we focus on the drug delivery systems engineered for SMO and GLI inhibitors as a valuable approach to improve their bioavailability and efficiency to cross the blood–brain barrier (BBB), one of the main challenges in the treatment of MB.

## Introduction

Medulloblastoma (MB) accounts for 15–20% of pediatric brain tumors, and it is a leading cause of cancer-related deaths in children. The current treatment for MB consists of surgery followed by craniospinal irradiation and chemotherapy, which includes vincristine, cisplatin, and cyclophosphamide or lomustine, depending on the patient’s age, suitability for radiotherapy, and risk category. This standard protocol often results in damages to the developing brain, especially in children under age three (Menyhárt and Győrffy, 2020). For this reason, the development of more effective and less toxic therapies has emerged as an opportunity to improve the prospect for MB patients.

During the past two decades, intensive molecular investigations have provided new insights into biology and clinical heterogeneity of MB. The latest classification recognizes four MB subgroups—wingless (WNT), sonic-hedgehog (SHH), group 3 (G3), and group 4 (G4)—each with different origins, genetic profiles, and variable prognoses, making the identification of a successful therapeutic strategy very difficult (Northcott et al., 2019; Hovestadt et al., 2020). Among MB variants, SHH-MB is the most prevalent one (∼30% of all MBs), and it is characterized by the aberrant activation of the Hedgehog (HH) signaling cascade, an evolutionary conserved pathway crucial for tissue development, regeneration, and stem cells maintenance (Briscoe and Thérond, 2013). The canonical activation of HH signaling occurs through the binding of the SHH ligand to the transmembrane receptor PATCHED 1 (PTCH1), which relieves the repression on the G protein-coupled Smoothened (SMO) co-receptor. This event initiates a complex intracellular cascade that leads to the dissociation of GLI transcription factors (GLI1, GLI2, and GLI3) from the cytoplasmic negative regulator suppressor of fused (SUFU) and to their following translocation into the nucleus. While GLI1 acts exclusively as a transcriptional activator, GLI2 and GLI3 can exert a repressor function in their cleaved forms (GLI2R and GLI3R) (Humke et al., 2010; Wang et al., 2010; Infante et al., 2018).

In response to SHH, the active full-length forms of GLI factors lead to the expression of their target genes involved in proliferation, angiogenesis, apoptosis suppression, and stemness (Ruiz i Altaba et al., 2002; Gulino et al., 2007; Traiffort et al., 2010). Mutations and copy-number variation in critical genes of HH signaling (i.e., *PTCH*, *SMO*, *SUFU*, and *GLIs*) cause an aberrant activation of this pathway and lead to formation of a wide spectrum of tumors (Skoda et al., 2018). Therefore, pharmacological blockade of HH signaling has emerged as a promising anticancer therapeutic approach and a number of HH inhibitors have been designed and developed (Lospinoso Severini et al., 2020). Most HH inhibitors affect the function of the SMO receptor even if their use for SHH-MB has shown several limits, especially due to SMO drug-resistance mutations (Wang et al., 2013; Atwood et al., 2015; Danial et al., 2016). Moreover, the existence of noncanonical SMO-independent mechanisms of activation of GLI transcriptional factors has brought out great interest in the discovery of molecules able to block the activity of GLI1, the most powerful and final effector of the HH pathway (Infante et al., 2015; Ghirga et al., 2018).

One of the main challenges in the development of successful SMO or GLI1 inhibitors for the treatment of MB is represented by their poor ability to cross the blood–brain barrier (BBB).

The structure of BBB, consisting of brain endothelial cells connected by tight junctions and covered by pericytes and basement membrane, limits the transport of many hydrophilic, protein-bound drugs, especially when their molecular weight exceeds 400 Da (Kadry et al., 2020). In this regard, nanoparticles, liposomes, or polymeric micelles carrying small molecules stand as promising tools to overcome this crucial issue, thus resulting in effective treatment of MB. In this minireview, we summarize recent progress in the development of drug delivery systems for SMO and GLI inhibitors overall based on the encapsulation of these compounds in nanoparticles, with particular focus on their use and efficacy on SHH-MB models.

### Drug Delivery Systems for SMO Antagonists

Significant progress has been made in the identification and synthesis of a broad class of SMO antagonists. Two of them, vismodegib (GDC-0449) and sonidegib (LDE225), have been approved by the Food and Drug Administration (FDA) for the treatment of metastatic or locally advanced basal cell carcinoma (BCC) and have entered in clinical trials for SHH-MB (Dlugosz et al., 2012; Pak and Segal, 2016; Rimkus et al., 2016; Casey et al., 2017; Lospinoso Severini et al., 2020). However, different toxicity profiles and SMO drug-resistance mutations have limited their advanced clinical investigation (Li et al., 2019). The major pitfalls of SMO inhibitors include both limited bioavailability and pharmacokinetics, due to unfavorable solubility, and low BBB permeability, thus restricting their use for central nervous system (CNS) tumors (Li et al., 2019; Lospinoso Severini et al., 2020; Dias et al., 2021). In this scenario, nanoparticle-based drug delivery systems represent a valid opportunity to overcome these issues, by improving the pharmacological properties and safety profile of SMO antagonists, as well as their delivery across BBB (Wei et al., 2014). To date, several drug delivery methods for the treatment of brain tumors have been exploited, including nanocarriers (viral vectors, nanoparticles, and exosomes), or noninvasive techniques, such as microbubble-enhanced diagnostic ultrasound (MEUS) (Dong et al., 2018; Haumann et al., 2020). In particular, the engineering and application of nanoparticles (NPs), successfully used for the treatment of different cancer types, has aroused great interest in ameliorating the bioavailability and BBB permeability of anticancer drugs for brain tumors, due to their ability to enter into the brain parenchyma (Lockman et al., 2002; Zhang et al., 2008).

NPs can be designed using a variety of materials, including lipids, polymers, metals, and inorganic particles. NPs carry drugs to tumors through three main strategies: i) passive targeting, which involves enhanced permeability and retention effect due to the poor vascular structure of the tumor microenvironment; ii) active targeting, through the functionalization or decoration of NPs with targeting moieties to promote internalization into tumor cells; iii) endogenous and/or exogenous stimuli-responsive targeting that triggers the drug release at the tumor site in a spatial–temporal control (Swetha and Roy, 2018; Manzari et al., 2021).

Currently, two different strategies have been proposed for the delivery of LDE225 and vismodegib in the treatment of SHH-MB: engineered HDL-mimetic (eHNPs) and poly(2-oxazoline) nanoparticles.

LDE225 (*N*-[6-[(2*S*,6*R*)-2,6-dimethylmorpholin-4-yl]pyridin-3-yl]-2-methyl-3-[4-(trifluoromethoxy)phenyl]benzamide) belongs to a class of biphenyl carboxamides and has been identified as a SMO antagonist able to bind its transmembrane domain (TMD) and to reduce tumor growth of both subcutaneous and subcortically orthotopic mice models implanted with Ptch^+/−^;p53^−/−^ MB cells. LDE225 was advanced into phase I clinical trials in 2010 and was approved by FDA in 2015 for treating locally advanced BCC (Pan et al., 2010).

**FIGURE 1:**
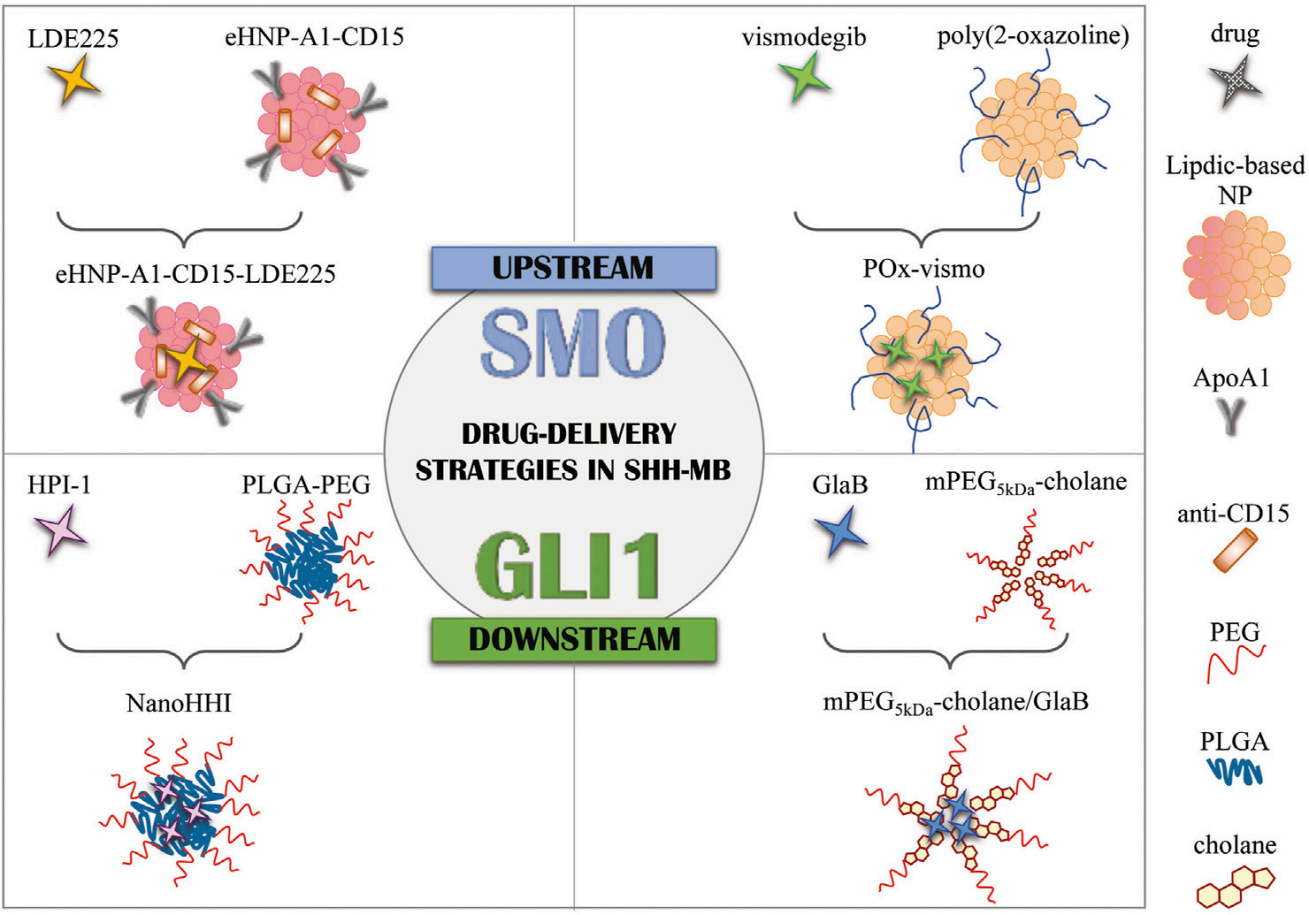
Drug delivery strategies in SHH-MB. Graphical representation of current formulation encapsulating SMO antagonists (LDE225 and vismodegib) and GLI1 inhibitors (HPI-1 and GlaB). NPs, nanoparticles; eHNP, engineered high-density lipoprotein-mimetic nanoparticle; ApoA1, apolipoprotein A1; PLGA, poly(lactic-*co*-glycolic acid; PEG, polyethylene glycol; mPEG_5kDa_-cholane: end-functionalization of 5 kDa linear amino-terminating monomethoxy-poly(ethylene glycol) (PEG-NH2) with 5β-cholanic acid; GlaB, glabrescione B.

Recently, Kim et al. (2020) employed engineered biomimetic high-density lipoprotein (HDL) nanoparticles (eHNPs) as an active targeting strategy to deliver LDE225 in SHH-MB (Table 1; Figure 1). The authors took advantage from the previous evidences that the uptake of HDL NPs in SHH-MB cells highly expressing the HDL receptor scavenger receptor class B type 1 (SR-B1), deprives cells of natural HDL and their cholesterol cargo, thereby blocking proliferation (Bell et al., 2018). Given that SHH signaling is regulated through cholesterol homeostasis, destroying the regulation of intracellular cholesterol could stand as an alternative therapeutic option to inhibit the activation of this pathway (Bidet et al., 2011; Ciepla et al., 2014; Huang et al., 2016; Xiao et al., 2017).

**TABLE 1:** Nanoformulations for brain drug delivery of small HH inhibitors. Table shows composition, *in vitro* and *in vivo* efficacy, size, and the BBB crossing ability of the nanoparticles for SHH-MB treatment under investigation.

Free drug	Target	NPs	DDS	*In vitro* Efficacy	*In vivo* Efficacy	Size (nm)	NP BBB crossing ability	Ref.
Sonidegib (LDE225) 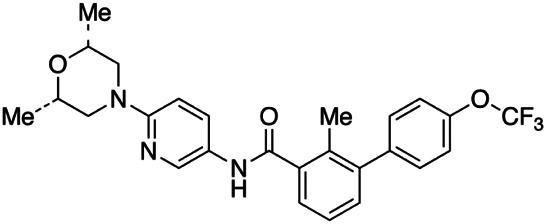	SMO	eHNP-A1-CD15 (DMPC, ApoA1, anti-CD15)	Liposomal nanoparticles	Cell viability inhibition DAOY; PZp53	HH-dependent MB growth inhibition and extended survival in SmoA1; Math-Cre-ER-Ptch^flox/flox^ mice	28	Detected in brain (24 h post i.v.)	Kim et al. (2020)
Vismodegib (GDC-0449) 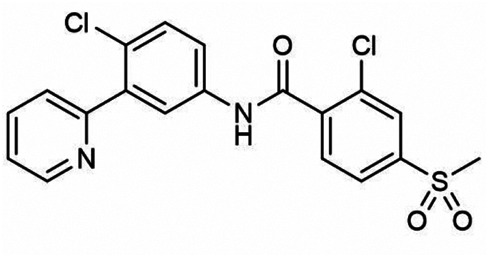	SMO	POx (polyoxazoline block copolymer)	Polymeric micelles	—	Reduction of free-drug systemic toxicity and extended survival in Healthy mice; Gfap-Cre/SmoM2 100 mg/kg	25–40	Not able	Hwang et al. (2020)
HPI-1 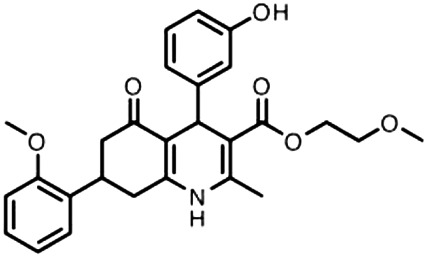	GLI1	NanoHHI (PLGA-PEG)	Polymer nanoparticles	—	HH-dependent MB growth inhibition in allograft model of primary MB cells from Smo^WT^/Smo^D477G^; Ptch^+/−^; Trp53^−/−^ mice 30 mg/kg	100	Detected in brain (3.9±2.1 mg/g 10’ post i.v.; 1.4 ± 0.4 mg/g 30’ post i.v.)	Chenna et al. (2012)
GlaB 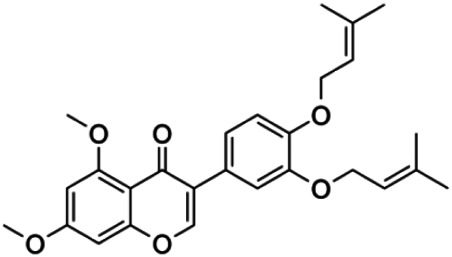	GLI1	mPEG_5kDa_-cholane	Polymeric micelles	Cell viability inhibition in primary MB cells from Math1-Cre/Ptc^C/C^ mice	HH-dependent MB growth inhibition and extended survival in allograft model of primary MB cells from Math1-Cre/Ptc^C/C^ mice 9 mg/kg	21.7 ± 0.7	Detected: in brain (1.93% ID/g 1 h post i.v.; 1.8% ID/g 2 h post i.v.) in cerebellum (1.87% ID/g 1 h post i.v.; 1.67% ID/g 2 h post i.v.)	Infante et al. (2021)

NPs, nanoparticles; DDS, drug delivery system; n.a., not available.

In addition to regulating biological processes, such as reverse cholesterol transport, HDL emerged also as a promising nanocarrier for targeted delivery of therapeutic molecules with high stability (Kuai et al., 2016). However, endogenous HDL purified from human plasma shows structural and functional heterogeneity, which may lead to unreproducible outcomes upon systemic administration for drug delivery (Fazio and Pamir, 2016). To this regard, Kim et al. (2020) used a microfluidic technology to reconstitute in a simple single-step eHNPs with high homogeneity and reproducibility for the targeted delivery and enhanced therapeutic efficacy of the SMO inhibitor, LDE225, in SHH MB cells. These nanoparticles were composed of apolipoprotein A1 (ApoA1), DMPC lipid (1,2-dimyristoyl-sn-glycero-3-phosphocho-line), anti-CD15 antibody, and LDE225 therapeutic cargo. ApoA1 and DMPC lipid form a nanoparticle shell that encapsulates the SMO inhibitor into a hydrophobic core. To enhance the delivery of LDE225 at the tumor site, the NPs have been designed for a “dual targeting” mediated by: i) the recognition of CD15 ligand expressed on murine SHH-MB cancer stem-like cells by the anti-CD15 antibody present on the surface of NPs; ii) the receptor-mediated transcytosis following the direct interaction of ApoA1 to SR-B1, expressed either on brain endothelial cells or SHH-MB cells (Kim et al., 2020). The effect on cell viability of each component in the proposed nanoparticles was tested in SHH MB cells (DAOY and PZp53) treated with free LDE225, eHNP-A1, eHNP-A1-CD15, eHNP-A1-LDE225, and eHNP-A1-CD15-LDE225. The treatment with eHNP-A1-LDE225 dramatically increased the therapeutic efficacy of free LDE225 (IC_50_ ∼70 nM and ∼2 μM, respectively). The addition of anti-CD15 in the NPs (eHNP-A1-CD15-LDE225) led to a drastic reduction of the IC_50_ value (∼8 nM). Interestingly, eHNP-A1 and eHNP-A1-CD15 without drug loading also showed therapeutic effects, reducing the cell viability of SHH-MB cells with a strength equivalent to a treatment concentration corresponding to 10 μM LDE225 (Kim et al., 2020). These observations support previous evidence regarding the inhibitory effects of HDL NPs on SHH-MB cells through the cholesterol efflux (Bell et al., 2018). The double mechanism of action of eHNP-A1-CD15-LDE225 offers the opportunity to maximize therapeutic outcome and to reduce the drug dosage (Kim et al., 2020). Importantly, eHNPs have shown the ability to cross the BBB and to target cancer stem-like cells both in *ex vivo* SmoA1 organotypic slice cultures and in *in vivo* SHH-MB mouse model (SmoA1^+/+^;Math1-GFP^+/+^). Intravenous (i.v.) administration of eHNP-A1-CD15-LDE225 increased the survival of SHH-MB mouse models (SmoA1-GFP and Patched knockout mice) and drastically impaired tumor growth by increasing apoptosis of tumor cells (Kim et al., 2020). Of note, these findings suggest eHNPs as a useful option for the delivery of other SMO-inhibitors that suffer of poor bioavailability and unable to cross the BBB.

A valid candidate in generating nanoparticle-based therapeutic products for tumor treatment is represented by the poly(2-oxazoline) amphiphilic block copolymer (POx). This drug delivery system, used for ovarian, breast, and lung cancers, has recently been described to optimize the clinical features of vismodegib (2-chloro-*N*-(4-chloro-3-(pyridin-2-yl)phenyl)-4-(methylsulfonyl)benzamide), the first FDA-approved inhibitor of SMO with low aqueous solubility and reduced bioavailability (Table 1; Figure 1) (Luxenhofer et al., 2010; Han et al., 2012; Hwang et al., 2020). Vismodegib is a benzamide obtained by formal condensation between the carboxy group of 2-chloro-4-(methylsulfonyl)benzoic acid and the anilino group of 4-chloro-3-(pyridin-2-yl). Vismodegib is able to bind the TMD of SMO and to induce tumor regression in a Ptch^+/-^ derived MB allograft mouse model (Robarge et al., 2009).

Hwang et al. (2020) used the thin film method to generate micelles with vismodegib:POx polymer (POx-vismo) in different ratios (2:10, 4:10, 6:10, and 8:10 w/w) (Hwang et al., 2020). The loading efficiency of vismodegib encapsulated in POx was nearly 90% and the loading capacity ranking from 13.5 to 42.4% w/w, depending on the drug:polymer ratio (Hwang et al., 2020). Intraperitoneal (i.p.) injection of POx-vismo (100 mg/kg) in MB-prone G-Smo mice revealed an increased pharmacokinetics compared to free vismodegib, solubilized with N-methyl-2-pyrrolidone (NMP) and PEG300. Higher concentration of vismodegib, released from POx-vismo micelles, was detected by liquid chromatography–mass spectrometry (LC-MS) in serum, MB, and forebrain until 8 h after administration, compared to free vismodegib. The analysis of exposure of tissues to the drug over time showed for the POx-vismo an improved uptake across BBB and limited distribution to nontarget organs, compared to free vismodegib. Similarly, the administration of POx-vismo in C57BL/6 mice with intact BBB increased CNS penetration, thus reducing systemic biodistribution (Hwang et al., 2020). Moreover, POx-vismo showed a limited toxicity effect on bone growth compared to free vismodegib in C57BL/6 mice treated at several post-natal days. Although, POx-vismo exhibited similar pharmacodynamic effects to systemically administer free vismodegib, this drug delivery system resulted to be more efficient to prolong survival in MB-prone G-Smo mice, with 30% of mice surviving to 35 days compared to the control group (Hwang et al., 2020).

These evidences underline as SMO antagonists encapsulated in NPs drug delivery system for the treatment of SHH-MB, could improve their ability to target the specific tumor site at optimal therapeutic concentration, thus mitigating their toxic effects. Moreover, the use of decorated NPs with additional components able to recognize specific antigens expressed on SHH-MB cells and cancer stem cells could offer the opportunity to increase the therapeutic effects on tumor cells, potentially avoiding off-target effects. Nevertheless, greater efforts need to be directed for the encapsulation of SMO antagonists in NPs for their advance in clinical practice.

### Drug Delivery Systems for GLI Inhibitors

In the field of research oncology for the treatment of SHH-MB, growing efforts have been focused on the development of small molecules acting as GLI inhibitors. Cancer cells can acquire resistance to SMO antagonists through secondary mutations in the SMO receptor, following drug administration (Yauch et al., 2009). Moreover, given that *GLI1* can be either primarily amplified or secondarily amplified/overexpressed in the setting of HH inhibitor therapy, a pharmacologic targeting of this transcription factor could have substantial benefits.

The most relevant contribution in the field of HH-driven tumor biology arises from the synthesis of GLI inhibitors. These compounds can act directly by blocking GLI transcriptional function or indirectly through the alteration of posttranslational modifications that control GLI activity (Infante et al., 2015).

In this minireview, we focus on GLI inhibitors whose delivery in *in vivo* MB models has been enhanced through encapsulation strategies.

In 2009, Hyman and colleagues identified a series of four small HH pathway inhibitors, namely HPI 1–4 (4-(3-Hydroxy-phenyl)-7-(2-methoxy-phenyl)-2-methyl-5-oxo-1,4,5,6,7,8-hexahydro-quinoline-3-carboxylic acid 2-methoxy-ethyl ester), which act by blocking GLI1 and GLI2 function through different mechanisms of action: HPI-1 targets posttranslational events of GLI processing/activation downstream of SMO; HPI-2 and HPI-3 alter the trafficking of GLI1 and increase the stability of GLI2; HPI-4 perturbs ciliogenesis by an unclear mechanism. Among them, only HPI-1 and HPI-4 have shown the ability to inhibit the proliferation of cerebellar granule neuron precursors (Hyman et al., 2009).

Although HPI-1 showed the highest efficacy in antagonizing both GLI1/GLI2 proteins compared to the other HPI inhibitors, its efficacy *in vivo* is hampered by highly lipophilic nature and poor aqueous solubility, thus impairing its systemic bioavailability. To overcome this drawback, HPI-1 has been encapsulated in a polymer nanoparticle (NanoHHI) using [poly(lactic-co-glycolic acid); (PLGA)] conjugated with polyethylene glycol (PEG) (Table1; Figure 1) (Chenna et al., 2012). *In vivo* studies performed in non-tumor bearing mice demonstrated the improvement of pharmacokinetic parameters and systemic bioavailability of HPI-1 encapsulated in the nanoformulation compared to the free drug, following both oral and parenteral administration. Of note, HPI-1 was readily detectable in brain tissue at 3.9 ± 2.1 mg/g at 10 min and 1.4 ± 0.4 mg/g at 30 min after single-dose intravenous administration. NanoHHI (30 mg/kg, i. p. administration) inhibited tumor growth in allograft models of MB derived from both Smo^WT^ and Smo resistant-mutant Smo^D477G^; Ptch^+/-^; Trp53^−/−^ mice, as consequence of the downregulation of *Gli1* gene expression (>50% compared to PLGA-PEG NPs used as control) (Chenna et al., 2012).

A consistent advance in the identification of GLI1 antagonists is represented by the synthesis of glabrescione B (GlaB) (3-(3,4′-bis(3-methylbut-2-enyloxy)phenyl)-5,7-dimethoxy-4H–chromen-4-one) as the first GLI1 inhibitor able to directly interact with the zinc-finger of this transcription factor, thus impairing the formation of the GLI1/DNA complex. GlaB is an isoflavone naturally found in the seeds of *Derris glabrescens* (*Leguminosae*) that inhibits the growth of HH-dependent tumors, including SHH-MB, both *in vitro* and *in vivo*, as well as the clonogenicity of cancer stem-like cells (Infante et al., 2015). However, the low aqueous solubility of GlaB (0.02 μg/ml) results in poor bioavailability, thus bringing out the need to improve this aspect in order to enhance its therapeutic efficacy.

To this regard, Ingallina et al. (2017) loaded GlaB into polymeric nanocapsules (NCs) composed of castor-oil-cored, thus increasing about 70-fold the aqueous solubility of this compound (∼700 μg GlaB/ml of NC). Good results have been obtained *in vitro* by serum stability assays showing a minimal drug release in blood circulation (<20% in 24 h), thus highlighting that most of the drug achieve the tumor site (Ingallina et al., 2017).

Recently, GlaB has been successfully encapsulated in a colloidal formulation of mPEG_5kDa_-cholane–based micelles, in order to ameliorate its solubility, biodistribution, ability to cross the BBB, and consequently its effectiveness in inhibiting SHH-MB growth (Table 1; Figure 1) (Infante et al., 2021).

mPEG_5kDa_-cholane is an amphiphilic polymer demonstrated to remarkably enhance the biopharmaceutical properties of either small or macromolecular drugs (Ambrosio et al., 2016). Compared to other amphiphilic polymers tested, mPEG_5kDa_-cholane yielded the highest GlaB solubility: 1.18 mM GlaB concentration was obtained with 0.4 mM of mPEG5_kDa_-cholane, corresponding to 26,000 fold GlaB concentration in water. GlaB-loaded micelles obtained with mPEG_5kDa_-cholane had a typical spherical micelle shape with a size of 16.9 ± 0.7 nm and a loading capacity of 27% w/w (Infante et al., 2021). Thanks to these properties, GlaB formulated in mPEG_5kDa_-cholane (mPEG_5kDa_-cholane/GlaB) affected the *in vitro* proliferation of primary SHH-MB cell cultures derived from Math1-cre/Ptc^C/C^ mice and significantly impaired GLI1 transcriptional activity compared to free GlaB (Infante et al., 2021). Promising data have also been reported *in vivo*: mPEG_5kDa_-cholane/GlaB administered with the dose of 9 mg/kg to nude mice grafted with primary HH-dependent MB cells strongly reduced the tumor growth rate and tumor volume more than GlaB dissolved at the same concentration in 2-HP-βCD/ethanol (3:1) or with cremophor/DMSO containing mixtures. Of note, HPLC coupled with electrospray mass spectrometry (HPLC-MS) analysis demonstrated the ability of mPEG_5kDa_-cholane/GlaB to cross the BBB and biodistribute into the brain and cerebellum of CD1 wild-type mice i.v. injected with the formulation (9 mg/kg) at different time points. In agreement with these results, i.v. administration of mPEG_5kDa_-cholane/GlaB drastically reduced tumor growth also in an orthotopic model of HH-dependent MB (Infante et al., 2021). These findings highlight that mPEG_5kDa_-cholane/GlaB is a promising candidate for clinical studies for the treatment of HH-dependent cancers and nowadays is the most encouraging drug delivery formulation for GLI inhibitors efficient in counteracting SHH-MB growth in preclinical investigation.

Little information is available for drug delivery systems of other GLI antagonists and for most of them there is no evidence of their efficacy in SHH-MB models.

Recently, epigenetic enzymes have emerged as druggable targets and critical regulators of HH transcriptional output. In particular, BRD4, a member of bromo and extra C-terminal (BET) bromodomain (BRD) proteins, is able to bind the promoter of GLI1 and GLI2, thus inducing their transcriptional activity. Tang and collaborators identified the small molecule JQ1 [(*S*)-(+)-*tert*-Butyl 2-(4-(4-chlorophenyl)-2,3,9-trimethyl-6*H*-thieno[3,2-*f*][1,2,4]triazolo[4,3-*a*][1,4]diazepin-6-yl)acetate] as a BRD4 inhibitor, capable of indirectly affecting the GLI1 activity and suppressing the tumor growth in several HH-dependent mouse models (BCC, MB, and atypical teratoid rhabdoid tumor) resistant to SMO antagonists (Tang et al., 2014). However, JQ1 is highly hydrophobic, a feature that hinders its delivery *in vivo*. A recent study investigated the anticancer activity of JQ1 encapsulated into apolipoprotein (ApoE) mimetic peptide decorated nanoparticles (ApoE-NPs) (Wang et al., 2020). In particular, this delivery strategy takes advantage of the mimetic ApoE peptide COG-133 consisting of only 18 amino acids sufficient to retain the binding potential to a very low-density lipoprotein (VLDL) receptor. This mimetic peptide has been conjugated with polymeric NPs to selectively target MB cells and achieve therapeutic concentration of JQ1 in the brain. In particular, cellular uptake of ApoE-NPs has been reported *in vitro* in both HD-MB03 (G3 MB) and DAOY (SHH-MB) cells showing a significant increase of targeted-NPs uptake than nontargeted NPs treatment. ApoE-NPs are specifically taken up by MB cells via the ligand-mediated endocytosis pathway. ApoE-NPs encapsulation improved JQ1 anticancer efficiency also *in vivo* as observed in orthotopic G3-MB bearing mice (10 mg/kg, systemic administration). ApoE-NPs formulation significantly enhanced JQ1 concentration in the brain and remarkably inhibited tumor cell proliferation and induces apoptosis (Wang et al., 2020).

Overall, these findings underline the relevance to further investigate efficient drug delivery systems for GLI antagonists, in order to ameliorate their biopharmaceutical properties and anticancer efficacy, thus accelerating their next clinical investigation for HH-dependent MB.

## Concluding Remarks

In the last decade, large-scale multi-omics analyses have confirmed the tight correlation between the hyperactivation of HH signaling and the MB tumorigenesis, leading to definition of the SHH-MB subgroup into four additional molecular subtypes according to the patient’s age and HH signaling gene alterations.

Although, the HH pathway emerged as one of the most attractive therapeutic target for this cancer entity, clinical applications of SMO antagonists are restricted because of their poor systemic bioavailability, development of drug-resistance mutations, and additional GLI activation via noncanonical pathways. Furthermore, the development of GLI inhibitors, as a valid alternative to overcome these pitfalls, is still limited due to their low pharmacological properties and BBB permeability.

Drug delivery to the brain represents one of the most important challenges in the field of CNS tumors, including MB. Indeed, astrocytes surrounding the BBB make it almost 98 and 100% impermeable to small and large molecules, respectively (Fischer et al., 1998). Interestingly, recent findings have shown that the MB genotype dictates the composition of the BBB and blood vessel phenotype. In particular, it was found that the WNT MB subtype has a better response to chemotherapy because it has a fenestrated vasculature, while non-WNT MB subtypes show an intact BBB, rendering them more resistant to chemotherapy (Phoenix et al., 2016). Moreover, during the development of brain malignancies, the cancer cells damage the BBB, leading to the formation of the tumor BBB (BBTB), another important physiological barrier to drug delivery to MB (Zhang et al., 1992).

At the light of these evidences, in recent years, drug delivery and nanomedicines have attracted significant attention for the treatment of MB. In particular, nanoparticles (NPs) offer concrete promise as carriers to enhance the delivery of small HH antagonists (Valcourt et al., 2020). As reviewed here, encapsulating SMO or GLI inhibitors inside NPs can improve their pharmacological properties, BBB permeability, and cell-specific delivery by coating NPs with ligands. This enables the nanoformulations much more effective than their freely delivered counterparts to suppress SHH-MB growth. Currently, only few SMO and GLI inhibitors encapsulated in drug delivery systems have been tested in *in vitro* and/or *in vivo* SHH-MB models. However, the promising results obtained emphasize how essential nanomedicine is for the development of safe and effective anticancer drugs for SHH-MB, in order to translate them into clinical practice. Finally, drug delivery systems also represent an opportunity for the improvement of combined targeted therapy in SHH-MB, since they facilitate the administration of synergistic drug combinations (Borah et al., 2020).

## Fundings

This study was funded by the Fondazione AIRC per la ricerca sul Cancro, Grant #IG20801, to LDM, Progetti di Ricerca di Università Sapienza di Roma, Italian Ministry of Health, Grant PRIN 2017BF3PXZ, to LDM, Pasteur Institute/Cenci Bolognetti Foundation to LDM, Dipartimenti di Eccellenza-L. 232/2016, Istituto Italiano di Tecnologia (IIT). LLS was supported by a FIRC-AIRC fellowship for Italy.
